# Evaluation of 2,7-Naphthyridines as Targeted Anti-Staphylococcal Candidates with Microbiota-Sparing Properties

**DOI:** 10.3390/ijms262110442

**Published:** 2025-10-27

**Authors:** Anna Wójcicka, Maciej Spiegel, Bartłomiej Dudek, Malwina Brożyna, Adam Junka, Marcin Mączyński

**Affiliations:** 1 Department of Organic Chemistry and Pharmaceutical Technology, Faculty of Pharmacy, Wroclaw Medical University, Borowska 211A, 50-556 Wroclaw, Poland; maciej.spiegel@umw.edu.pl (M.S.); marcin.maczynski@umw.edu.pl (M.M.); 2 Platform for Unique Model Application, Division of Translative Technologies, Wroclaw Medical University, Borowska 211, 50-556 Wroclaw, Poland; bartlomiej.dudek@umw.edu.pl (B.D.); malwina.brozyna@umw.edu.pl (M.B.); adam.junka@umw.edu.pl (A.J.)

**Keywords:** 2,7-naphthyridines, antimicrobial activity, cytotoxicity, molecular dynamics simulations

## Abstract

The rising resistance of bacterial and fungal strains, particularly in biofilm form, is diminishing the efficacy of available therapies and poses a major threat to human health. This highlights the need for new antimicrobial agents. A review of biological studies has shown that 2,7-naphthyridine derivatives exhibit a wide spectrum of pharmacological properties, including antimicrobial activity, which has contributed to the development of new compounds containing this scaffold. In this work, the obtained compounds were tested to assess their ability to eradicate biofilm formed by selected reference strains of opportunistic pathogens: *Staphylococcus aureus*, *Pseudomonas aeruginosa* and *Candida albicans* as well as towards normal microbiota representative, referred to as the *Lactobacillus crispatus*. The tested 2,7-naphthyridine derivatives showed selective antimicrobial activity, exclusively against *S. aureus*. **10j** demonstrated the highest, among tested compounds, activity on this pathogen (MIC = 8 mg/L), while compound **10f** exhibited ~100-fold stronger activity (MIC = 31 mg/L) than the majority of the library compounds. The in vitro assessment on fibroblast cell lines demonstrated low cytotoxicity of both compounds **10f** and **10j**, which was subsequently confirmed in vivo using the *Galleria mellonella* larval model, where no signs of systemic toxicity were observed during the 5-day observation period. Due to the structural similarity of the compounds **10f** and **10j** to typical gyrase/topoisomerase IV inhibitors, molecular dynamics simulations were performed on a ternary complex containing protein, DNAds, and a 1,5-naphthyridine inhibitor (PDB ID: 6Z1A). Molecular dynamics of the gyrase–DNA ternary complex supported stable binding of both hydrazone derivatives, with **10j** showing slightly more favorable MM/GBSA energetics driven by electrostatics and halogen bonding, consistent with its ~4-fold lower MIC versus **10f**. Taken together, our data highlight compound **10j** as a promising microbiota-sparing antibacterial candidate, particularly suitable for selective interventions against *S. aureus*, for instance in vaginal infections, where targeted eradication of the pathogen without disturbing protective commensals is highly desirable.

## 1. Introduction

Biofilm is a complex aggregation of microorganisms embedded in an extracellular polymeric matrix that significantly enhances microbial tolerance to chemical and physical agents [1]. This protective barrier limits the efficacy of many therapeutic strategies and contributes to the persistence of infections, especially in clinical settings. In this context, there is a growing demand for the development of novel compounds capable of disrupting biofilms and overcoming the limitations of existing antimicrobial therapies, including antiseptics and conventional antibiotics. Recent discoveries have also emphasized the pivotal role of host-associated microbiota in maintaining local immunity and mucosal health [2]. Disruption of these commensal communities, particularly in anatomically and immunologically distinct environments such as the vaginal niche, has been linked to increased infection susceptibility, inflammation, and treatment failure [3]. As such, next-generation antimicrobial agents should ideally combine efficacy against target pathogens with a high degree of selectivity, sparing the resident microbiota. Antiseptics, while effective, are inherently broad-spectrum and typically act without distinction between beneficial and harmful microorganisms [4]. This highlights the need for new agents that exert focused antimicrobial activity without undermining the physiological microbial equilibrium.

The vaginal environment represents a particularly relevant model for evaluating the selectivity of such antimicrobial agents. In healthy individuals, this niche is typically dominated by a stable and low-diversity microbiota, composed primarily of *Lactobacillus* species such as *L. crispatus*, *L. jensenii*, and *L. gasseri*, which contribute to mucosal homeostasis and protection against pathogens [5]. Disruption of this microbial balance, whether due to antibiotic use, hormonal changes, or exogenous infections, can be predisposed to symptomatic conditions. These include fungal overgrowth, most caused by *Candida albicans*, as well as bacterial vaginosis (BV), which may involve overrepresentation of *Gardnerella vaginalis*, anaerobic Gram-negative bacilli, or Gram-positive pathogens such as *Staphylococcus aureus* [6]. As such, therapeutic approaches targeting vaginal infections must ideally preserve beneficial lactobacilli while eliminating disease-associated microorganisms.

This therapeutic gap has prompted interest in small-molecule scaffolds with tunable physicochemical properties and diverse biological activity. Among such structures, *N*-heterocyclic compounds continue to occupy a central position in drug discovery due to their synthetic versatility and well-documented pharmacological potential [7,8,9,10].

Naphthyridines are *N*-heterocyclic compounds consisting of two fused pyridine rings and can exist in six positional isomers depending on the placement of nitrogen atoms.

The first naphthyridine derivative introduced into medical use as an antibacterial agent was nalidixic acid [11].

In the subsequent years, numerous other naphthyridine-based compounds demonstrating antimicrobial properties were synthesized, with several entering clinical practice, including amfonelic acid, enoxacin, tosufloxacin, gemifloxacin, zabofloxacin, trovofloxacin, and alatrofloxacin [12]. Nalidixic acid and its derivatives exhibit structural similarity to quinolones, a large group of antibacterial compounds. Anti-microbially active compounds contain a carbonyl group at the 3-position of the quinoline or naphthyridine system [13,14,15].

Type II topoisomerase (DNA gyrase) is crucial for deoxyribonucleic acid (DNA) replication and transcription. Due to its essential role in bacterial survival, it is an attractive target for antimicrobial drugs [16]. The development of new inhibitors of this enzyme is crucial in combating the growing threat of antibiotic resistance [17]. Binding of nalidixic acid derivatives to the gyrase/topoisomerase IV-DNA complex inhibits DNA replication, which accounts for their bacteriostatic and bactericidal properties [18].

Derivatives of the isomer 2,7-naphthyridine exhibit a broad spectrum of biological activity, notably including antimicrobial effects [19]. This pharmacological versatility has made the 2,7-naphthyridine scaffold an attractive starting point for the development of novel therapeutic agents [20].

**Figure 1:**
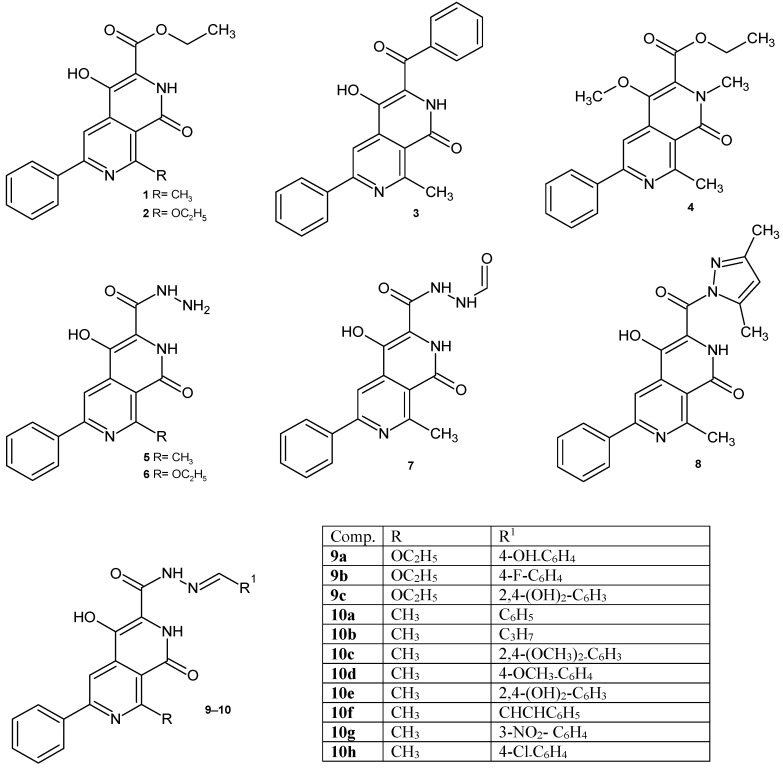
2,7-Naphthyridine derivatives studied in this work.

**Figure 2:**
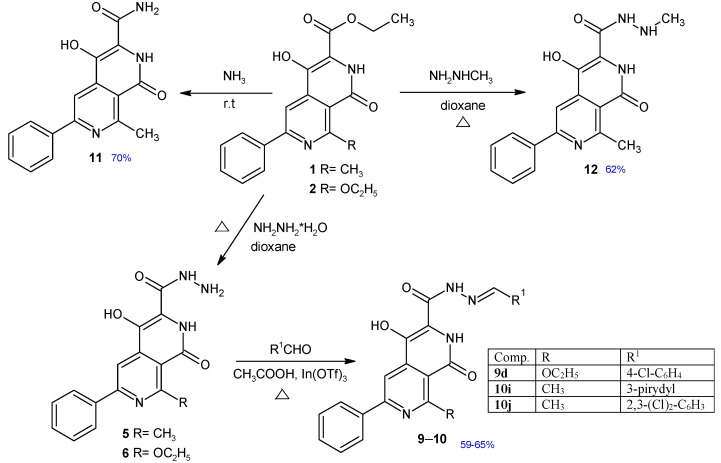
Synthesis of new 2,7-naphthyridine derivatives **9**–**12**. Chemical yields of new compounds are shown in blue.

Based on the above data, we decided to investigate the antimicrobial activity of the synthesized 2,7-naphthyridine derivatives containing a carboxyl group at position 3 and their potential binding to DNA gyrase. In our previous papers, the methods of synthesis 2,7-naphthyridines were described [21,22,23]. Continuing our work on the biological activity of 2,7-naphthyridines, it was decided to test the compounds obtained in previous years (Figure 1), as well as several newly synthesized derivatives **9d**, **10i–j**, **11**, **12** (Figure 2), for their potential antimicrobial/antibiofilm activity.

In this work, the obtained compounds were tested to assess their ability to eradicate biofilm formed by selected reference strains of opportunistic pathogens: *S. aureus*, *P. aeruginosa* and *C. albicans* as well as towards normal vaginal microbiota representative, referred to as the *Lactobacillus crispatus.*

Also, given the need to evaluate both antimicrobial efficacy and host-directed cytotoxicity, we adopted a two-tiered experimental strategy. As a first-line model, murine fibroblasts were used for in vitro cytotoxicity screening. Although not tissue-specific, fibroblast lines represent a well-established and sensitive platform for preliminary toxicological assessment, offering a pragmatic proof-of-concept approach at early development stages [24]. To complement these findings, we employed the *G. mellonella* larval model, which is increasingly recognized surrogate for early-phase pharmaco-toxicological studies. To our knowledge, this is the first report utilizing *G. mellonella* to investigate the biological activity of 2,7-naphthyridine derivatives. Owing to its compartmentalized physiology, including hemolymph-based circulation, epithelial barrier analogues, and conserved innate immune responses, this invertebrate model provides a valuable intermediate step between cell-based assays and vertebrate systems [25]. Accordingly, we evaluated a selection of 2,7-naphthyridine derivatives for their antimicrobial activity, cytotoxic profile, and in vivo tolerability, with particular interest in their potential applicability to mucosal (f.e. vaginal) infections, requiring selective microbial targeting.

## 2. Results

### 2.1. Chemical Synthesis

The synthesis and characterization of compounds **1**, **2**, **3**, **4**, **5**, **6**, **7**, **8**, **9a**–**c, 10a**–**h** have been published previously [21,22,23]. A series of new 2,7-naphthyridine derivatives were synthesized according to the procedure shown in Figure 2. Ethyl 4-hydroxy-8-methyl-1-oxo-6-phenyl-1,2-dihydro-2,7-naphthyridine-3-carboxylate **1** and ethyl 8-ethoxy-4-hydroxy-1-oxo-6-phenyl-1,2-dihydro-2,7-naphthyridine-3-carboxylate **2** obtained earlier [23] were found to be useful as key intermediates for the further synthesis of other 2,7-naphthyridine derivatives. The synthesis of new Schiff bases **9d** and **10h–j** involved the reaction between appropriate aldehydes and hydrazides **5** or **6** in a presence of catalytic amount of indium (III) trifluoromethanesulfonate. The amide **11** was obtained in the reaction of ester **1** with ammonia solution. Treatment of ester **1** with methylhydrazine resulted in the formation of 4-hydroxy-N’,8-dimethyl-1-oxo-6-phenyl-1,2-dihydro-2,7-naphthyridine-3-carbohydrazide **12**. Structures of new compounds and their spectral analysis are shown in the Supplementary Material (Figures S1–S20).

### 2.2. Evaluation of Biological Effects Displayed by Obtained Compounds

As a first-line screening, the antimicrobial activity of selected 2,7-naphthyridine derivatives was evaluated against *S. aureus*, *P. aeruginosa* and *C. albicans* strains. Among the tested microorganisms, only *S. aureus* exhibited a measurable response, with compound **10j** demonstrating a MIC and MBC equal of 8 mg/L and compound **10f** with respective values of MIC and MBC equal of 31 mg/L. No MIC was detected for *P. aeruginosa* or *C. albicans*, even at the highest applied concentration (1000 mg/L), suggesting a selective antimicrobial effect limited to Gram-positive cocci (Table 1). To enable comparison with a clinically used antiseptic agent, a 0.1% polyhexanide-containing formulation was tested under identical conditions; the results are presented in Table S1 shown in the Supplementary Material.

**Figure 3:**
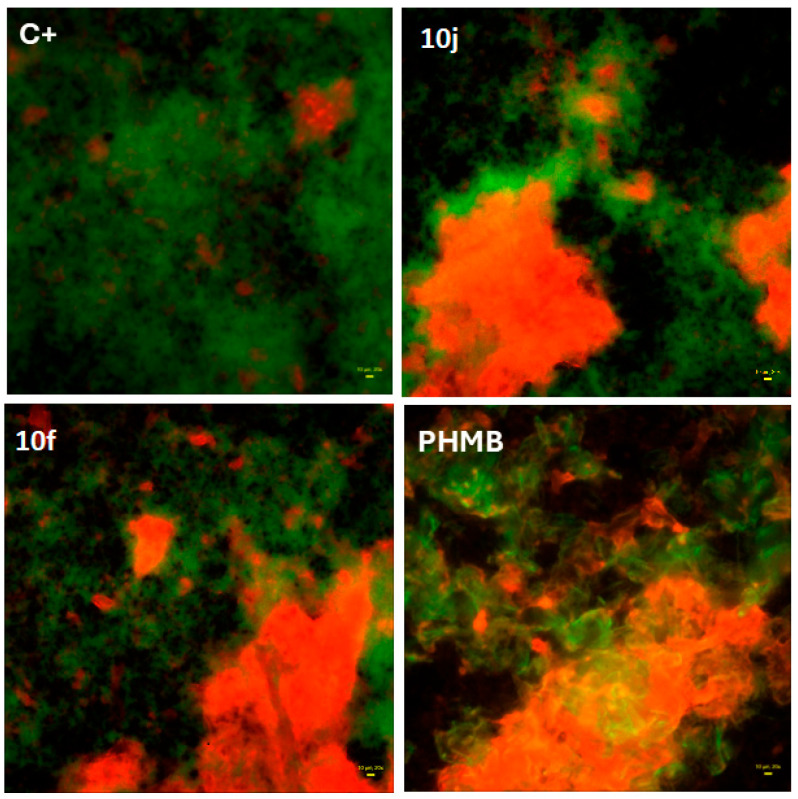
Fluorescence-based live/dead staining of *S. aureus* biofilm exposed to **10j** (8 mg/L) or **10f** (31 mg/L) compounds compared to controls: (positive one, C+: consisted of saline, while for control of method’s utility, polyhexanide (PHMB) was used as the reference antiseptic. Red fluorescence indicates dead cells; green fluorescence indicates live cells. Images were acquired using the Live/Dead Biofilm Viability Kit (Thermo Fisher Scientific Inc., Waltham, MA, USA) and 20× objective, LumaScope 620 microscope. Yellow rectangle in lower right part of images represents 20 µm of length.

To further assess potential anti-biofilm properties, fluorescence-based live/dead imaging was performed using *S. aureus* biofilms exposed to the most promising compounds **10j** and **10f**. As shown in Figure 3, both induced a marked increase in the proportion of dead bacterial cells (red fluorescence) compared to the saline control (C+).

The observed pattern was comparable to the effect of polyhexanide (PHMB), a clinically approved antiseptic used here as a reference standard, supporting the compound’s capacity to disrupt preformed biofilm structures.

**Figure 4:**
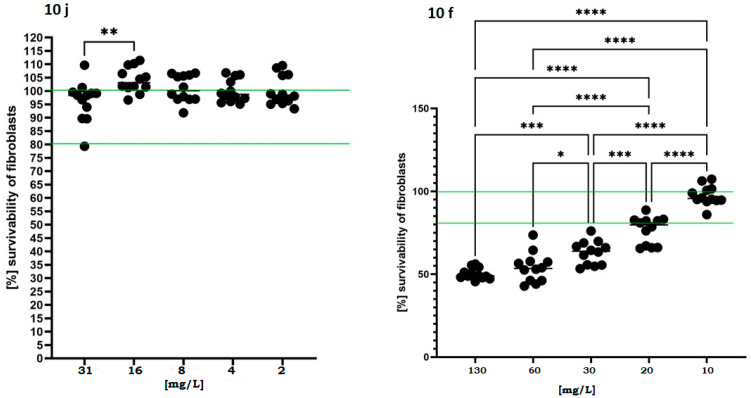
Viability of murine fibroblasts (L929) following 24 h exposure to **10j** and **10f** compounds. Cell viability was measured using a colorimetric assay after treatment with increasing concentrations of each compound (2–130 mg/L). Results are expressed as mean ± standard deviation, normalized to untreated control (100%). The dashed horizontal line at 100% represents baseline viability of untreated cells, while the second line at 80% denotes the commonly accepted cytotoxicity threshold below which a compound is considered to exert a biologically relevant toxic effect. Statistically significant differences versus control are indicated by asterisks: *p* < 0.05 (*), *p* < 0.01 (**), *p* < 0.001 (***), *p* < 0.0001 (****), based on one-way ANOVA followed by Dunnett’s post hoc test.

In parallel, the cytotoxic potential of the active compounds was assessed using fibroblasts exposed to a concentration gradient exceeding MIC/MBC values recorded for compounds **10j** and **10f**. As shown in Figure 4, the fibroblast viability remained above 80% across all tested concentrations, and in certain conditions even exceeded 100% relative to untreated controls in case of compound **10j**.

This effect may suggest a proliferative or protective response induced at subtoxic concentrations. In contrast, compound **10f** induced a more pronounced dose-dependent decline in viability, dropping below 60% at 30 mg/L. This data suggests that compound **10j** displays a favorable safety profile and a potentially broader therapeutic window in future translational applications.

**Figure 5:**
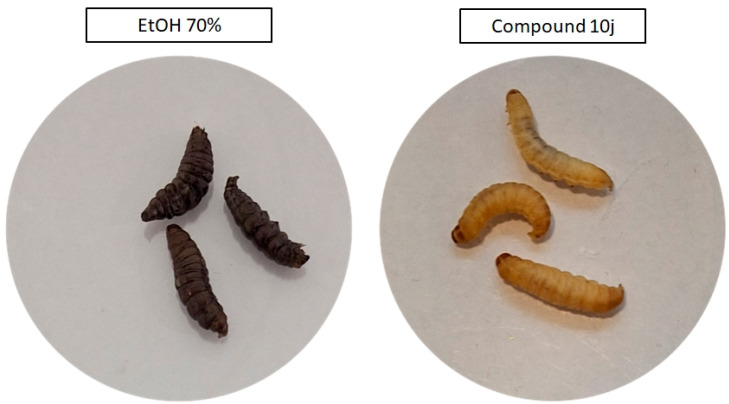
Representative images *of Galleria mellonella* larvae 24 h after injection with **10j** compound (right site of image panel), or 70% ethanol (positive control, left site of image panel). Larvae treated with **10j** compound retain normal coloration, turgor, and motility, with no signs of melanization or distress. In contrast, ethanol-exposed larvae exhibit dark melanization and loss of structural tone, indicative of acute toxicity.

Following the in vitro assessment on fibroblasts, the in vivo tolerability of the selected compounds was evaluated using the *G. mellonella* larval model. Both **10j** and **10f** were administered at concentrations corresponding (due to solubility issues) to those used in cytotoxicity testing, and no signs of systemic toxicity were observed over the 5-day observation period. All larvae maintained normal motility, pigmentation, and survival rates, indicating good biocompatibility of both compounds. In contrast, the positive control group exposed to 70% ethanol displayed rapid melanization and complete mortality within 24 h post-injection, confirming the assay’s sensitivity to cytotoxic effects and validating the non-toxic profile of the tested derivatives under the applied conditions (Figure 5).

Given the observed selective antibacterial activity and the favorable cytotoxicity profile of **10j** and **10f** compound, their potential for application in microbiota-sensitive environments was further explored also. To this end, both compounds were evaluated against *Lactobacillus crispatus*, which is a key representative of the healthy vaginal microbiota. MIC, MBC, and MBEC values were determined using the standard microdilution and biofilm eradication assays. Neither **10j** nor **10f** exhibited bacteriostatic, bactericidal, or biofilm-disruptive activity against *L. crispatus* at concentrations up to 1000 mg/L, suggesting that both compounds are microbiota-sparing and thus potentially suitable for targeted antimicrobial interventions that preserve beneficial commensal bacteria. Also, to verify whether the antimicrobial activity of the most promising compound **10j** extends beyond the initially tested Gram-positive *S. aureus* and Gram-negative *P. aeruginosa*, two additional reference strains were included—*Staphylococcus epidermidis* (Gram-positive) and two strains of *Escherichia coli* (Gram-negative). The results obtained using the 96-well microdilution method confirmed the previous observations: compound **10j** exhibited a MIC/MBC of 3.9 mg/L against the Gram-positive coccus, whereas no inhibitory effect was observed against the Gram-negative rod within the tested concentration range up to 1000 mg/L.

### 2.3. Molecular Modelling

The computational results indicate that the tested substances effectively bind within the designated site, as evidenced by the Molecular mechanics/Generalized-Born surface area (MM/GBSA) computations (Table 2). The highest binding affinity was observed for **10j**, as indicated by its Kd of 1.37 × 10^−3^ M^−1^. However, the binding constants remain statistically indistinguishable between the binding poses and between the two compounds, as the differences are marginal. Nevertheless, the experimentally observed lower MIC/MBC values for **10j** compared to **10f** are consistent with the computational findings.

A detailed analysis of the binding contributions suggests that the differences in binding affinity arise primarily from electrostatic interactions between the two compounds and variations in polar solvation energies between the two conformers. **10j** is more strongly favoured over **10f** in terms of Coulomb interactions, while the β orientation is slightly preferred over the α orientation, albeit still positive in energy contribution.

To rationalize these observations, it is important to consider the binding environment. On one side, the ligands are enclosed within a highly electronegative region due to the dissociated phosphate groups. On the other side, the binding pocket consists of two aspartate residues and, deeper within, four methionines, creating another negatively charged region. The presence of two chlorine atoms attached to the aromatic system of **10j** appears to mitigate unfavourable interactions in two ways: (I) by pulling electron density toward themselves, inducing a local dipole moment and a hydrophobic character that complements the electronegative regions, and (II) by forming halogen bonds with adjacent oxygen or nitrogen atoms in the DNA bases. Additionally, the shorter –CH_2_– linkage between the diazo bond and phenolic rings in **10j** compared to **10f** likely prevents excessive insertion into the pocket, thereby reducing overlap between the negatively charged electron clouds of the amino acids and the ligand. Regarding the preference for the β orientation, this highlights the importance of the naphthyridine rings being positioned between the two negatively charged regions. A parallel, bidirectional synthetic strategy, optimizing ligand interactions with local positively charged sites, could potentially enhance binding affinity further. However, additional factors are likely involved. These include differences in intracellular accumulation and permeability through the Gram-positive envelope, susceptibility to efflux, solubility and aggregation behaviour in broth, nonspecific binding to media components, binding kinetics (residence time) versus equilibrium affinity, compound protonation states, and microenvironmental factors at the intracellular target site.

The absence of activity against *P. aeruginosa* and *C. albicans* further underscores the selective profile of **10j**. In *P. aeruginosa*, resistance is likely multifactorial, involving limited outer membrane permeability, active efflux, and potentially reduced target accessibility or affinity. The compound’s physicochemical features, such as moderate lipophilicity and halogenation, may hinder its accumulation in this intrinsically drug-resistant species. In the case of *C. albicans*, the absence of antifungal activity is not unexpected, given the structural divergence between fungal and bacterial topoisomerases, which likely precludes effective binding. Moreover, the hydrazone scaffold lacks known antifungal properties, and no fungicidal effect was observed even at the highest test concentrations. These findings support the view that **10j** does not act as a broad-spectrum agent but rather exerts selective antibacterial activity with particular relevance for *S. aureus*.

## 3. Discussion

The growing resistance of pathogens to antibiotics and antiseptics underscores the urgent need for new antimicrobial agents with selective activity and low cytotoxicity. Existing drugs often display potent but non-specific effects, damaging host cells or disrupting the protective microbiota. Therefore, current research increasingly focuses on compounds that can effectively eradicate pathogens while preserving eukaryotic cell viability and microbial homeostasis. Within this framework, naphthyridines represent a particularly promising scaffold. Their tunable electronic and steric properties allow the design of derivatives with targeted antimicrobial activity and improved safety margins [26]. Building on this rationale, we evaluated a panel of 2,7-naphthyridine derivatives for their antibacterial selectivity, cytotoxicity, and molecular interactions with bacterial targets. The results revealed a narrow but distinct activity spectrum, limited to Gram-positive cocci, with negligible or no inhibition of *P. aeruginosa*, *C. albicans*, or *L. crispatus* Among all tested molecules, compounds **10f** and **10j** exhibited the strongest antibacterial effects, with compound **10j** showing the lowest minimal inhibitory and bactericidal concentrations (Table 1). This selective efficacy toward Gram-positive *Staphylococci* (but not Gram-negative rods) combined with the absence of detectable toxicity toward mammalian fibroblasts (Figure 4) and in the *G. mellonella* in vivo model (Figure 5) indicates a promising therapeutic window. Such a profile is particularly desirable for applications targeting infections in microbiota-sensitive niches, where traditional, non-selective antiseptics are often too aggressive [27]. Mechanistically, these findings prompted a deeper investigation into the relationship between structure and antibacterial effect. Since the observed activity was restricted to *Staphylococcus* spp. and exhibited a clearly bactericidal pattern, we focused on hypothesizing which molecular features determine this selectivity and potency. The antimicrobial profile of the synthesized 2,7-naphthyridine derivatives demonstrates a remarkable degree of selectivity and potency toward *S. aureus*, with compound **10j** standing out as the most active member of the series. The parallel determination of MIC and MBC values revealed that, for this strain, both parameters were identical (MIC = MBC = 8 mg/L), satisfying the CLSI (Clinical and Laboratory Standards Institute) criterion for a bactericidal mode of action (MBC/MIC ≤ 4) [28]. Live/Dead fluorescence imaging (Figure 3) further confirmed this observation, showing a rapid predominance of propidium-iodide-positive cells after 1 h exposure- an effect visually comparable to that produced by the reference antiseptic polyhexanide (PHMB). These results indicate that **10j** induces irreversible bacterial death rather than transient growth inhibition. Such bactericidal behaviour is consistent with its predicted binding to DNA gyrase, a classical target of lethal topoisomerase-poisoning mechanisms, and distinguishes it from agents that merely halt replication [29]. The structure–activity relationship (SAR) analysis suggests that antibacterial potency originates primarily from the 2,7-naphthyridine scaffold in combination with the hydrazone linker (-C=N–NH-), which together constitute the essential pharmacophoric core. The planar, conjugated system of 2,7-naphthyridine allows π–π stacking with nucleic acid bases and aromatic residues within the gyrase–DNA complex, while the hydrazone bridge maintains conjugation and contributes hydrogen-bond donors and acceptors necessary for stable binding. Substituent effects on the terminal aryl ring may further modulate the interaction: electron-withdrawing groups, particularly halogens, enhance electrostatic complementarity and may correlate with lower MIC values. The dichlorophenyl substituent of **10j** not only improves binding affinity via halogen bonding and dipolar interactions but may also stabilize the complex through improved hydrophobic matching within the negatively charged environment of the gyrase pocket, as supported by molecular dynamics (MD) and MM/GBSA analyses (Table 2). By contrast, other analogues lacking these optimal features were biologically inactive, despite close structural resemblance. Electron-donating or sterically bulky substituents near the hydrazone bond may disturb molecular planarity and electronic distribution, weakening π–π stacking and hydrogen bonding within the enzyme pocket [30,31]. Additionally, these modifications likely reduced cell permeability, particularly across the double-membrane architecture of Gram-negative bacteria. The absence of detectable activity against *P. aeruginosa* and *E. coli* can thus be attributed to the combined effects of low compound accumulation, efflux, and decreased target accessibility, whereas in *C. albicans* the lack of effect reflects fundamental differences between bacterial (prokaryotic) and fungal (eukaryotic) topoisomerases. Overall, these findings illustrate how small deviations in molecular geometry and charge distribution can abolish activity in this compound class. Comparison with the clinically used antiseptic polyhexanide (Prontosan^®^, 0.1% PHMB) provides an additional pharmacological reference point. PHMB exhibited low MIC/MBC values across both Gram-positive and Gram-negative bacteria (1–4 mg/L) but also demonstrated partial activity toward *L. crispatus* (Table S1). In contrast, **10j** maintained potent killing of *S. aureus* and *S. epidermidis* while sparing *L. crispatus* and all Gram-negative and fungal species, highlighting its microbiota-sparing profile. This degree of selectivity is uncommon among antiseptic agents and suggests that **10j** may offer a therapeutic advantage in microbiota-sensitive environments, such as the vaginal mucosa, where preservation of commensal lactobacilli is critical for maintaining mucosal homeostasis [2]. From a mechanistic standpoint, the bactericidal activity of **10j** likely arises from two complementary processes: (i) stable inhibition of DNA gyrase leading to accumulation of double-strand breaks and replication arrest, and (ii) membrane disruption reflected by rapid Propidium Iodide uptake in biofilm assays. Such dual action—topoisomerase poisoning reinforced by membrane perturbation is characteristic of highly effective antibacterial scaffolds. The strong correlation between the electrostatically favourable halogen-bonding pattern observed in MD simulations and the four-fold lower MIC compared with **10f** further supports a target-driven mechanism. Together, these data position **10j** as a structurally defined, selective, and bactericidal candidate with promising translational potential. Despite the encouraging outcomes, several limitations of this study should be acknowledged. The antimicrobial assays were conducted exclusively on reference laboratory strains, which do not fully represent the phenotypic and genetic diversity of clinical isolates. Consequently, the selective activity observed against Gram-positive cocci should be interpreted cautiously until validated on a broader clinical panel. Moreover, the explanation for this selectivity, derived from molecular dynamics and physicochemical analyses, remains hypothetical and requires experimental verification through bacterial uptake and target-binding studies. Another limitation concerns the chemical behaviour of the investigated molecules: their moderate solubility and stability in aqueous environments may have influenced apparent potency and restricted achievable concentrations in the in vivo larval assay, potentially underestimating the therapeutic window. Additionally, while molecular modelling strongly suggests interaction with bacterial DNA gyrase, direct enzymatic validation of this mechanism has not yet been performed. When compared to the clinically established antiseptic PHMB, the lead compound **10j** exhibited lower absolute potency but a more selective and microbiota-sparing activity profile, a combination rarely found among existing antiseptics. This distinction emphasizes its potential as a next-generation scaffold for the development of safer, more targeted antimicrobials. Taken together, these findings delineate both the promise and the current boundaries of this research. Further studies should expand testing to clinical isolates, assess in-depth the mechanism of Gram-positive selectivity, and explore structural optimization to improve solubility and bioavailability. Although preliminary, the present results establish a solid conceptual foundation for the rational design of selective, non-cytotoxic, bactericidal agents derived from the 2,7-naphthyridine scaffold. In summary, the present study highlights 2,7-naphthyridine derivatives—particularly compound **10j**—as promising leads combining bactericidal efficacy with structural selectivity, offering a rational starting point for the development of safer and microbiota-preserving antimicrobial agents.

## 4. Materials and Methods

### 4.1. Chemistry

Commercially available chemicals for the syntheses were purchased from Chempur (Piekary Slaskie, Poland) or Sigma-Aldrich (Merck Group, Darmstadt, Germany) and were used without purification. The course of the reactions and the purity of the obtained compounds were checked by thin-layer chromatography (TLC) using aluminium sheet Silica gel 60 F254 (Merck KGaA, Darmstadt, Germany) and visualized by ultraviolet light at 254 or 365 nm using CAMAG UV Lamp 4 (CAMAG, Muttenz, Switzerland). Melting points were determined by MEL-TEMP apparatus (Barnstead International, Dubuque, IA, USA) and were uncorrected.

IR spectra were performed on the Thermo Scientific Nicolet iS50 FT-IR spectrometer (Thermo Fisher Scientific Inc., Waltham, MA, USA) using the ATR technique. FT-IR spectra analyses were performed using Omnic Spectra 2.0 software (Thermo Fisher Scientific Inc.).

Electrospray Ionization Mass Spectroscopy (ESI–MS) spectra were performed on the compact™ Bruker Daltonic Electrospray Ionization-Quadrupole-Time of Flight (ESI-Q-TOF) apparatus (Bruker Daltonik GmbH, Bremen, Germany). ESI-MS were conducted in positive-ion and/or negative-ion mode, and LC-MS-grade methanol was used as a solvent. ESI-MS spectra analyses were performed using Bruker Compass Data Analysis 4.2 software (Bruker Daltonik GmbH, Bremen, Germany).

^1^H and ^13^C NMR spectra were recorded in deuterated dimethyl sulfoxide (DMSO-*d*_6_) or deuterated chloroform (CDCl_3_) with TMS as the internal standard using the following instruments: Bruker Avance ARX-300 MHz spectrometer (Bruker Analytic, Karlsruhe, Germany) or Bruker NMR AVANCE III™ 600 MHz spectrometer with Ascend™ technology magnet (Bruker Corporation, Billerica, MA, USA). NMR spectral analyses were performed using MestReNova (Mnova version 14.2) software (Mestrelab Research S.L., Santiago de Compostela, Spain).

The NMR, IR, MS and elemental microanalyses were performed in the Laboratory of Elemental Analysis and Structural Research, Faculty of Pharmacy, Wroclaw Medical University.

**General Procedure for Preparation of Schiff’s bases** (**9–10**)

To a solution of hydrazide **5** or **6** (0.01 mol) in 20 mL of glacial acetic acid the appropriate aldehyde (0.01 mol) and catalytic amount of indium (III) trifluoromethanesulfonate (1.6 × 10^−6^ mol) were added. The mixture was heated under reflux with stirring for 4–6 h. After cooling, the precipitate was collected. Recrystallization from toluene afforded the title compounds **9**–**10**. Spectral analysis data of new compounds are shown in the Supplementary Material (Figures S1–S12).

*8-Ethoxy-4-hydroxy-1-oxo-6-phenyl-1,2-dihydro-2,7-naphthyridine-3-carboxylic acid (4-chloro-bezylidene)-hydrazide* (**9d**)

This compound was obtained as beige solid in 61% yield; m.p. = 269–271 °C.

ATR-FTIR υ max (cm^−1^): 3350, 3300 (NH), 2950 (CH), 1600 (C=N), 1530 (N-H), 1360 (OH).

^1^H NMR (300 MHz, CDCl_3_) δ (ppm): 1.61–1.66 (t, 3H *J =* 8.0 Hz, CH_3_), 4.91–4.95 (q, 2H *J* = 6.0 Hz, CH_2_), 7.20 (s, 1H, CH), 7.38–7.40 (m, 1H, Ar), 7.47–7.53 (m, 4H, Ar), 7.76–7.81 (m, 3H, Ar), 8.18–8.19 (m, 1H, Ar), 8.49 (s, 1H, Ar), 10.97 (s, 2H, OH/NH), 12.16 (s, 1H, NH).

^13^C NMR (300 MHz, DMSO-*d*_6_) δ (ppm): 14.47, 63.38, 96.06, 106.25, 108.86, 116.27, 116.42, 124.76, 127.14, 127.46, 127.53, 128.06, 128.81, 130.68, 131.26, 132.25, 132.92, 136.91, 141.16, 158.88, 163.07, 165.62, 167.32, 190.53

ESI-MS: *m/z* calculated for formula C_24_H_19_ClN_4_O_4_: [M − H]^−^ 461.1022; found: 461.1212.

Elemental Analysis calculated for formula C_24_H_19_ClN_4_O_4_: C, 62.27; H, 4.14; N, 12.10%; Found: C, 62.07; H, 4.23; N, 11.64%.

*4-Hydroxy-8-methyl-1-oxo-6-phenyl-N’-[(pyridin-3-yl)methylidene]-1,2-dihydro-2,7-naphthyridine-3-carbohydrazide* (**10i**)

This compound was obtained as beige solid in 59% yield; m.p. = 259–260 °C.

ATR-FTIR υ max (cm^−1^): 3220 (NH), 1630 (C=N), 1550 (N-H), 1310 (OH).

^1^H NMR (300 MHz, DMSO-*d*_6_) δ (ppm): 3.10 (s, 3H, CH_3_), 7.51 (s, 1H, CH), 7.52–7.58 (m, 3H, Ar), 8.16–8.21 (m, 4H, Ar), 8.23–8.27 (m, 1H, Ar), 8.46 (s, 1H, Ar), 8.66 (s, 1H, Ar), 8.93 (s, 1H, OH), 12.17 (s, 1H, NH).

^13^C NMR (300 MHz, DMSO-*d*_6_) δ (ppm): 22.49, 111.26, 115.33, 126.75, 127.52, 128.06, 128.15, 128.48, 128.76, 128.95, 129.10, 129.40, 130.63, 131.37, 137.79, 137.97, 138.14, 142.56, 155.01, 155.41, 165.96, 167.11.

ESI-MS: *m/z* calculated for formula C_22_H_17_N_5_O_3_: [M + H]^+^ 400.1404; found: 400.1378; [M − H]^−^ 398.1258; found: 398.1398.

*N’-[(2,3-dichlorophenyl)methylidene]-4-hydroxy-8-methyl-1-oxo-6-phenyl-1,2-dihydro-2,7-naphthyridine-3-carbohydrazide* (**10j**)

This compound was obtained as yellow solid in 65% yield; m.p. = 293–295 °C.

ATR-FTIR υ max (cm^−1^): 3320 (NH), 1680 (C=N), 1540 (N-H), 1350 (OH).

^1^H NMR (300 MHz, DMSO-*d_6_*) δ (ppm): 3.09 (s, 3H, CH_3_), 7.52–7.57 (m, 3H, Ar), 7.79 (s, 1H, CH), 8.15–8.20 (m, 3H, Ar), 8.29–8.31 (m, 2H, Ar), 8.52 (s, 1H, Ar), 8.75 (s, 1H, OH), 10.63 (s, 1H, NH), 12.66 (s, 1H, NH).

^13^C NMR (300 MHz, DMSO-*d*_6_) δ (ppm): 22.49, 111.26, 115.36, 126.73, 127.28, 127.45, 127.55, 128.17, 128.27, 128.55, 128.76, 128.95, 129.43, 129.61, 130.63, 137.84, 138.90, 139.07, 142.65, 155.01, 155.32, 165.88, 167.06.

ESI-MS: *m/z* calculated for formula C_23_H_16_Cl_2_N_4_O_3_: [M+H]^+^ 467.0672; found: 467.0680; [M−H]^−^ 465.0527; found: 465.0700.

Elemental Analysis calculated for formula C_23_H_16_Cl_2_N_4_O_3_: C, 59.11; H, 3.45; N, 11.99%; Found: C, 59.37; H, 3.47; N, 12.06%.

**Synthesis of** *4-Hydroxy-8-methyl-1-oxo-6-phenyl-1,2-dihydro-2,7-naphthyridine-3-carboxamide* (**11**)

To 50 mL solution of ammonia 3.54 g ester **1** (0.01 mol) was added. The mixture was stirred for 10 h. The precipitate was collected. Recrystallization from ethanol afforded the title compound **11** as beige solid in 70% yield; m.p. = 255–257 °C. Spectral analyses of the obtained compound are shown in the Supplementary Material (Figures S13–S16).

ATR-FTIR υ max (cm^−1^): 3400 (NH_2_), 3220 (NH), 1690 (C=N), 1560 (N-H).

^1^H NMR (300 MHz, DMSO-*d*_6_) δ (ppm): 3.09 (s, 3H, CH_3_), 7.52–7.63 (m, 2H, Ar), 7.76–7.78 (m, 1H, Ar), 8.16–8.28 (m, 3H, Ar), 8.50 (s, 1H, OH), 8.75 (s, 1H, NH), 11.70 (s, 1H, NH).

^13^C NMR (300 MHz, DMSO-*d*_6_) δ (ppm): 20.84, 110.94, 113.03, 116.85, 122.09, 127.41 (2C), 128.90 (2C), 130.46, 137.19, 142.42, 155.56, 160.88, 167.85, 169.31.

ESI-MS: *m/z* calculated for formula C_16_H_13_N_3_O_3_: [M+H]^+^ 296.1029; found: 296.1027.

**Synthesis of** *4-Hydroxy-N’,8-dimethyl-1-oxo-6-phenyl-1,2-dihydro-2,7-naphthyridine-3-carbohydrazide* (**12**)

To a solution of ester **1** (0.01 mol) in 100 mL of anhydrous dioxane, 2 mL of 98% methylhydrazine (0.02 mol) was added. The mixture was heated under reflux with stirring for 3 h. After cooling, the precipitate was collected. Recrystallization from methanol afforded the title compound **12** as brown solid in 62% yield; m.p. = 346–348 °C. Spectral analysis of the obtained compound **12** are shown in the Supplementary Material (Figures S17–S20).

ATR-FTIR υ max (cm^−1^): 3300 (NH), 2900 (OH), 1610 (C=N), 1590 (N-H).

^1^H NMR (300 MHz, DMSO-*d*_6_) δ (ppm): 1.98 (s, 3H, CH_3_), 3.08 (s, 3H, CH_3_), 7.51–7.58 (m, 3H, Ar), 8.21–8.24 (m, 2H, Ar), 8.27 (s, 1H, Ar), 10.45 (s, 1H, OH), 10.54 (br, 2H, NH).

^13^C NMR (300 MHz, DMSO-*d*_6_) δ (ppm): 15.68, 37.68, 110.78, 119.42, 122.64, 122.75, 129.74 (2C), 131.73 (2C), 134.54, 134.95, 138.06, 141.81, 154.30, 164.37.

ESI-MS: *m/z* calculated for formula C_17_H_16_N_4_O_3_: [M + H]^+^ 325.1295; found: 325.1252.

### 4.2. Computational Methods

All-atom molecular dynamics simulations were performed using *Amber22* [32] to investigate the inhibitory and antibacterial potential of the active substance against *S. aureus* (ATCC 25923). Given the structural similarity of the synthesized compounds to typical gyrase/topoisomerase IV inhibitors, the simulations were conducted on a ternary complex comprising the protein, the DNAds, and a 1,5-naphthyridine inhibitor (PDB ID: 6Z1A) [33].

**Figure 6:**
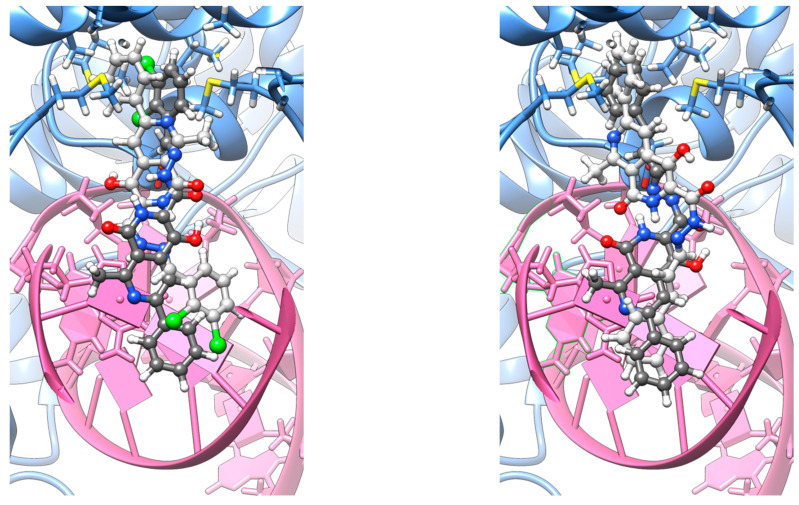
Representative docking poses **10j** (left) and **10f** (right), depicted as a superposition of α (dim gray) and β (light gray) orientations.

Initially, all co-crystallized small molecules were removed from the structure, and the protein was protonated following the PROPKA methodology [34,35,36]. Subsequently, the **10j** and **10f** ligands were manually inserted into two distinct orientations—denoted as α and β (Figure 6)— reflecting the structural symmetry of each molecule’s termini. In both cases, the ligand extends from the intercalation site toward the binding pocket located at the GyrA α3 helices [37].

Molecular dynamics simulations were performed using the following protocol. Ligand parameters were generated using a RESP-fitting procedure [38,39] implemented in the *antechamber* and *parmchk2* tools (AmberTools, University of California, San Francisco, USA)with the GAFF2 force field [40] applied to the ligand. In contrast, the protein and DNA were treated with the ff19SB [41] and OL15 [42] force fields, respectively. The entire complex was then solvated in a 15.0 Å box of OPC water [43], filled with K^+^ and Cl^−^ ions to neutralize the system and achieve a physiological salt concentration.

The system underwent stepwise energy minimization, during which harmonic restraints on the ligand, amino acids, and nucleotides were gradually released. This was followed by a two-step heating process—first to 100 K and subsequently to 303 K—over a total duration of 210 ps. Equilibration was conducted in three phases: 1 ns with backbone restraints, 1 ns with Cα restraints, and 1 ns without restraints (this equilibration step was repeated 10 times). The production run was then carried out for 100 ns. Throughout the simulations, the SHAKE algorithm [44] was applied to constrain bonds involving hydrogen atoms. Temperature was controlled using a Langevin thermostat with a collision frequency of 2.0 ps^−1^, and pressure was maintained via a Monte Carlo barostat. The simulation outputs were analyzed using the CPPTRAJ (v6.24.0) utilities [45].

Binding energetics were evaluated using the Molecular Mechanics Generalized Born Surface Area (MM/GBSA) [46] method across the entire simulation, with an implicit ionic strength of 0.150 M to match the simulation conditions. Topologies required for binding free energy calculations were generated using the *ante-MMPBSA* script, and the analysis was performed with the *MMPBSA.py* code [47]. Binding constants (in M) were computed using Equation (1):

K_d_ = e^ΔG/RT^ (1)

where ΔG is the binding energy (J mol^−1^), R is the gas constant (J mol^−1^ K^−1^), and T is the temperature in Kelvin (298 K, assuming standard conditions).

### 4.3. Biological Assessments

The following reference strains and eukaryotic cell line from ATTC (Manassas, VA, USA) were scrutinized: *S. aureus* 29213, *P. aeruginosa* 115442, *C. albicans* 10231, *L. crispatus* 33197, *Staphylococcus epidermidis* 12228, *Escherichia coli* 10536, *Escherichia coli* 8739 and L929 fibroblasts.

#### 4.3.1. Determination of Minimum Inhibitory and Bactericidal Concentrations (MIC and MBC)

The antimicrobial activity of the tested 2,7-naphthyridine derivatives was evaluated against tested microbes using the broth microdilution method, in accordance with the Clinical and Laboratory Standards Institute (CLSI) guidelines, with minor modifications. Compounds were dissolved in DMSO and serially diluted two-fold in appropriate growth media to achieve final concentrations ranging from 1000 mg/L to 1.95 mg/L. For *S. aureus* and *P. aeruginosa*, Mueller–Hinton Broth (MHB) was used; for *C. albicans*, RPMI 1640 medium buffered with MOPS; and for *L. crispatus*, de Man, Rogosa and Sharpe (MRS) broth was employed. The final concentration of DMSO did not exceed 1% (*v*/*v*) and had no inhibitory effect on microbial growth. Standardized inocula (5 × 10^5^ CFU/mL) were added to each well in 96-well plates to a final volume of 200 µL and incubated under strain-appropriate conditions: 24 h at 37 °C for bacteria, 48 h at 35 °C for *C. albicans*, and 24 h at 37 °C under microaerophilic conditions provided by GasPack for *L. crispatus*. MIC was defined as the lowest concentration at which no visible microbial growth was observed. For MBC determination, two methods were employed in the parallel spot method: 10 µL samples from wells without visible growth were spotted onto appropriate agar plates (Mueller–Hinton, Sabouraud, or MRS) and incubated for 24–48 h; dilution regrowth method: 100 µL of the same non-turbid well was transferred into 9.9 mL of fresh medium and incubated under the same conditions for 24 h. The absence of visible growth confirmed bactericidal/fungicidal activity. MBC was defined as the lowest concentration at which no regrowth was observed in either method. All assays were performed in triplicate in three independent experiments. The same methodology was applied for commercially applied antiseptic agent containing polyhexanide (Prontosan^®^ Wound Irrigation Solution; B. Braun Melsungen AG, Melsungen, Germany; 0.1% polyhexanide [polyhexamethylene biguanide hydrochloride, PHMB] and 0.1% undecylenamidopropyl betaine as active ingredients, with 0.5% glycerol and purified water as excipients.)

#### 4.3.2. Live/Dead Fluorescence Imaging of *S. aureus* or *L. crispatus* Biofilm

To assess the effect of selected compounds on preformed Staphylococcus aureus biofilms, a fluorescence-based viability assay was conducted using the Live/Dead™ BacLight™ Bacterial Viability Kit (Thermo Fisher Scientific, Waltham, MA, USA), according to the manufacturer’s instructions. Biofilms were established by inoculating 5 × 10^5^ CFU/mL of *S. aureus* ATCC 6538 into 96-well flat-bottom polystyrene plates containing 200 µL of Mueller–Hinton Broth per well. The plates were incubated at 37 °C for 24 h under static conditions to allow biofilm formation. After incubation, planktonic cells were gently aspirated, and the wells were washed twice with sterile phosphate-buffered saline (PBS) to remove non-adherent bacteria. The remaining biofilms were then exposed to test compounds (**10j** and **10f**) at concentrations corresponding to MIC and MBC values. Polyhexanide (PHMB) was used as a positive control, and sterile saline as a negative control. Treatments were applied for 1 h at 37 °C, followed by a gentle PBS wash. Biofilms were then stained with a mixture of SYTO 9 and propidium iodide dyes and incubated for 15 min in the dark at room temperature. Fluorescence was visualized using an inverted fluorescence microscope (e.g., Olympus IX73) equipped with appropriate filters. Green fluorescence (SYTO 9) indicates live bacteria with intact membranes, while red fluorescence (PI) indicates membrane-compromised, dead cells. Images were acquired at 20× magnification using Etaluma Lumascope 620 microscope (Etaluma Inc., San Diego, USA). Representative fields from each treatment were selected based on fluorescence distribution and biofilm density.

#### 4.3.3. Cytotoxicity Assessment on Fibroblasts

The cytotoxic potential of the selected compounds was evaluated in vitro using murine fibroblast cell line L929 (ATCC CCL-1), in accordance with ISO 10993-5 guidelines for the biological evaluation of medical devices. Cells were cultured in Dulbecco’s Modified Eagle Medium (DMEM; Sigma-Aldrich, St. Louis, MO, USA) supplemented with 10% fetal bovine serum (FBS) and 1% penicillin–streptomycin and maintained at 37 °C in a humidified atmosphere of 5% CO_2_. For the assay, fibroblasts were seeded into 96-well plates at a density of 1 × 10^4^ cells per well and allowed to adhere for 24 h. The medium was then replaced with fresh medium containing the test compounds (**10j** or **10f**) at serial concentrations ranging from 2 to 31 mg/L. Cells were incubated for an additional 24 h. After treatment, cytotoxicity was assessed using the neutral red uptake assay, according to the standard protocol. Absorbance was measured using a microplate reader at 540 nm for NR. Cell viability was calculated as a percentage relative to untreated control cells (set as 100%). A viability below 80% was considered indicative of cytotoxic potential. All treatments were performed in triplicate and repeated in three independent experiments.

#### 4.3.4. In Vivo Toxicity Assay in *Galleria mellonella*

The in vivo toxicity of compounds **10j** and **10f** was assessed using the *Galleria mellonella* larval model, following the protocol described by Dudek et al. [48], with slight modifications. Final-instar larvae (weighing 200–250 mg) were randomly assigned to groups of 10 individuals per condition. Each larva was injected with 10 µL of the test solution into the last left proleg using a Hamilton syringe (model 701 RN, 10 µL) fitted with a 26-gauge needle. Compound **10f** was administered at its MBC concentration (31 mg/L), while compound **10j** was tested at ¼ MBC (8 mg/L), due to technical limitations related to increased solution viscosity that interfered with injection. Two control groups were included: a positive control (70% ethanol) to confirm the sensitivity of the model, and a negative control (0.9% NaCl) to account for mechanical trauma and handling. Following injection, larvae were incubated at 30 °C in the dark and monitored daily for five days. Survival was determined based on the absence of movement in response to gentle tactile stimulation. Additional observations included melanization and changes in external morphology. All experiments were repeated in triplicate. Damaged or nonviable larvae prior to injection were excluded from analysis.

## 5. Conclusions

A series of 2,7-naphthyridine derivatives containing a carboxyl group at position 3 were synthesized and evaluated for antimicrobial, antibiofilm, and cytotoxic properties. Among them, Schiff base derivatives, particularly **10f** and **10j**, showed potent and selective bactericidal activity against *S. aureus*, while remaining inactive toward *P. aeruginosa* and *C. albicans*. Compound **10j**, bearing a dichloro-substituted phenyl ring, exhibited the strongest activity (MIC = 8 mg/L) and demonstrated significant disruption of *S. aureus* biofilm, comparable to the reference antiseptic polyhexanide (PHMB). Importantly, both **10f** and **10j** displayed low cytotoxicity toward fibroblasts and in the *G. mellonella* model, indicating a favorable therapeutic window. Molecular dynamics simulations supported their stable interaction with bacterial DNA gyrase, consistent with the bactericidal mode of action observed experimentally.

Taken together, these results identify compound **10j** as a promising microbiota-sparing antibacterial lead combining potent antibiofilm activity, low cytotoxicity, and selective action against *S. aureus*. Future studies should optimize its solubility and evaluate efficacy against clinical isolates and complex biofilm models to advance this compound toward translational application.

## Figures and Tables

**Table 1:** Antimicrobial activity of 2,7-naphthyridine derivatives (compounds **1**–**12**) determined by the microdilution method. MIC, MBC—minimal inhibitory concentration, minimal biocidal concentration, respectively; “non-detected”—no MIC/MBC found in tested range of compounds’ concentrations. Green-fonts indicate compounds and their concentrations applied for subsequent analyses.

Compounds	MBC [mg/L]						
	5	6	9a	10a	10f	10j	PHMB
*P. aeruginosa*	-	-	-	-	-	-	4
*S. aureus*	500	250	250	500	30	7.8	2
*C. albicans*	-	-	-	-	-	-	8

**Table 2:** MM/GBSA energy components (in kcal mol^−1^).

ΔG_C–R–L_	10j		10f	
Component	α	β	α	β
Van der Waals	−63.0	−56.6	−58.3	−56.6
Electrostatic Energy	0.1	−20.2	5.0	−9.1
Polar Solvation Energy	25.3	38.4	22.3	36.1
Non–Polar Solvation Energy	−6.1	−6.1	−6.0	−6.6
Σ	−43.7	−44.4	−37.0	−36.1
Binding constant	1.62 × 10^−3^	1.37 × 10^−3^	5.31 × 10^−3^	6.25 × 10^−3^

## Data Availability

All necessary data are presented in the manuscript and the raw data. can be provided by the authors upon reasonable request.

## Supplementary Materials

The following supporting information can be downloaded at: https://www.mdpi.com/article/10.3390/ijms262110442/s1.
