# Supplementary material for: Evaluation of 2,7-Naphthyridines as Targeted Anti-Staphylococcal Candidates with Microbiota-Sparing Properties
Source: Int J Mol Sci. 2025 Oct 27;26(21):10442. doi: 10.3390/ijms262110442 (PMC12609412; doi:10.3390/ijms262110442)
Supplement: Supplementary file 1 [file ijms-26-10442-s001.zip › ijms-3873705-supplementary.pdf]

SUPPLEMENTARY MATERIAL

Evaluation of 2,7-Naphthyridines as Targeted Anti-Staphylococcal Candidates with Microbiota-Sparing Properties

Anna Wójcicka [1,*], Maciej Spiegel [1], Bartłomiej Dudek[2], Malwina Brożyna[2], Adam Junka[2] and Marcin Mączyński [1]

[1] Department of Organic Chemistry and Pharmaceutical Technology, Faculty of Pharmacy, Wroclaw Medical University, Borowska 211A, 50-556 Wroclaw, Poland
[2] Platform for Unique Model Application, Division of Translative Technologies, Wroclaw Medical University, Borowska 211, 50-556 Wroclaw, Poland
* Correspondence: anna.wojcicka@umw.edu.pl;

Figure S1. ^{1}H NMR spectrum of compound **9d** ... 3

Figure S2. ^{13}C NMR spectrum of compound **9d** ... 3

Figure S3. ESI-MS spectrum of compound **9d** ... 4

Figure S4. FT-IR spectrum of compound **9d** ... 4

Figure S5. ^{1}H NMR spectrum of compound **10i** ... 5

Figure S6. ^{13}C NMR spectrum of compound **10i** ... 5

Figure S7. ESI-MS spectrum of compound **10i** ... 6

Figure S8. FT-IR spectrum of compound **10i** ... 6

Figure S9. ^{1}H NMR spectrum of compound **10j** ... 7

Figure S10. ^{13}C NMR spectrum of compound **10j** ... 7

Figure S11. ESI-MS spectrum of compound **10j** ... 8

Figure S12. FT-IR spectrum of compound **10j** ... 8

Figure S13. ^{1}H NMR spectrum of compound **11** ... 9

Figure S14. ^{13}C NMR spectrum of compound **11** ... 9

Figure S15. ESI-MS spectrum of compound **11** ... 10

Figure S16. FT-IR spectrum of compound **11** ... 10

Figure S17. ^{1}H NMR spectrum of compound **12** ... 11

Figure S18. ^{13}C NMR spectrum of compound **12** ... 11

Figure S19. ESI-MS spectrum of compound **12** ... 12

Figure S20. FT-IR spectrum of compound **12** ... 12

Table S1. Antimicrobial activity of Prontosan® ... 13

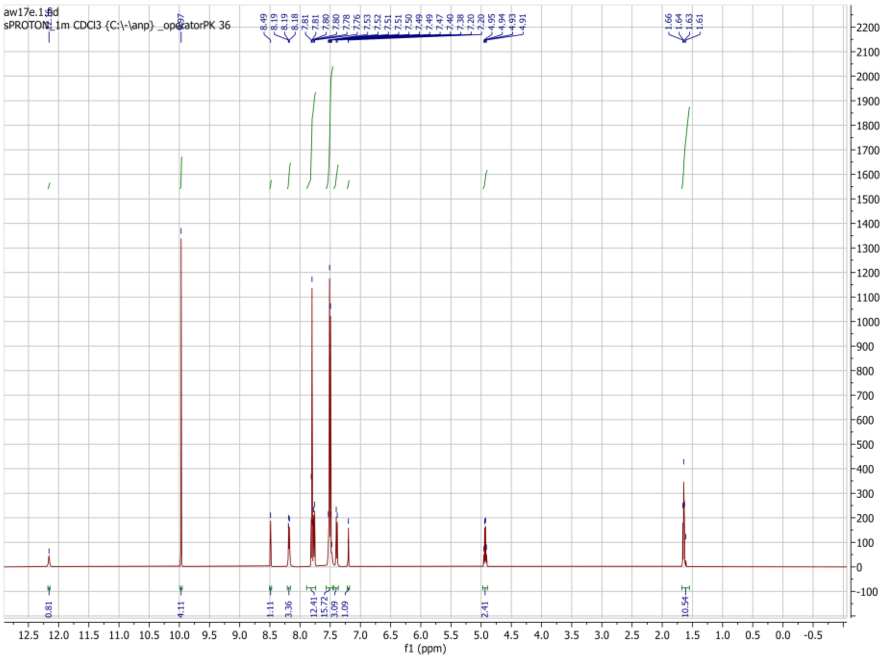

Figure S1. ^{1}H NMR spectrum of compound **9d**

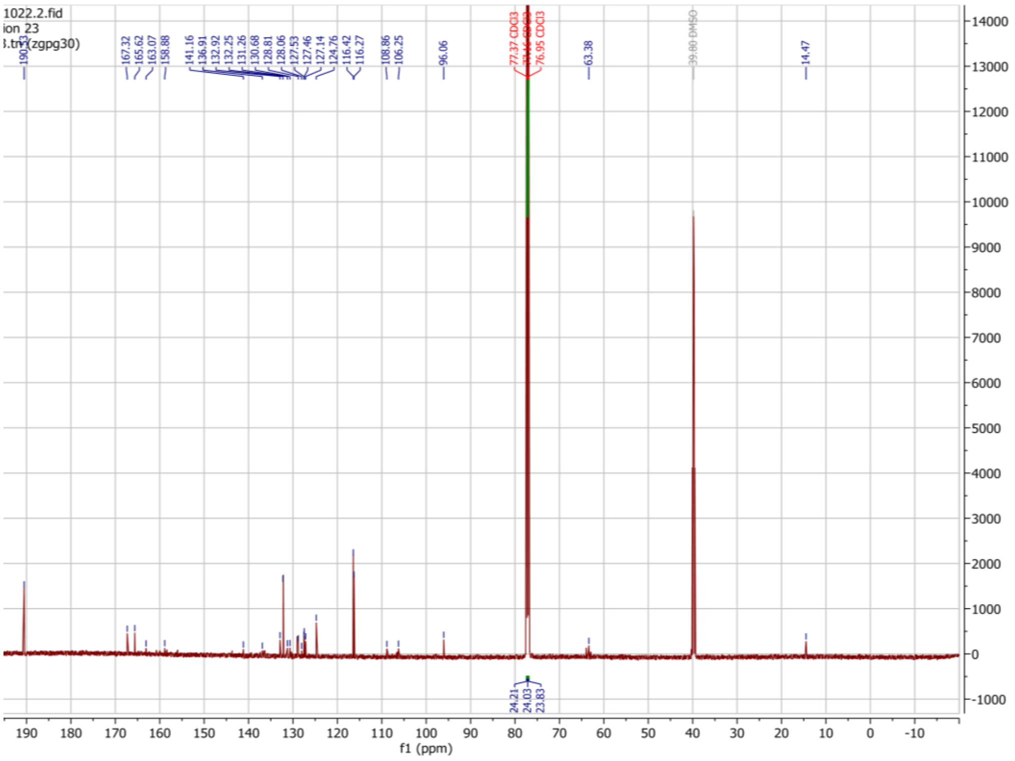

Figure S2. ^{13}C NMR spectrum of compound **9d**

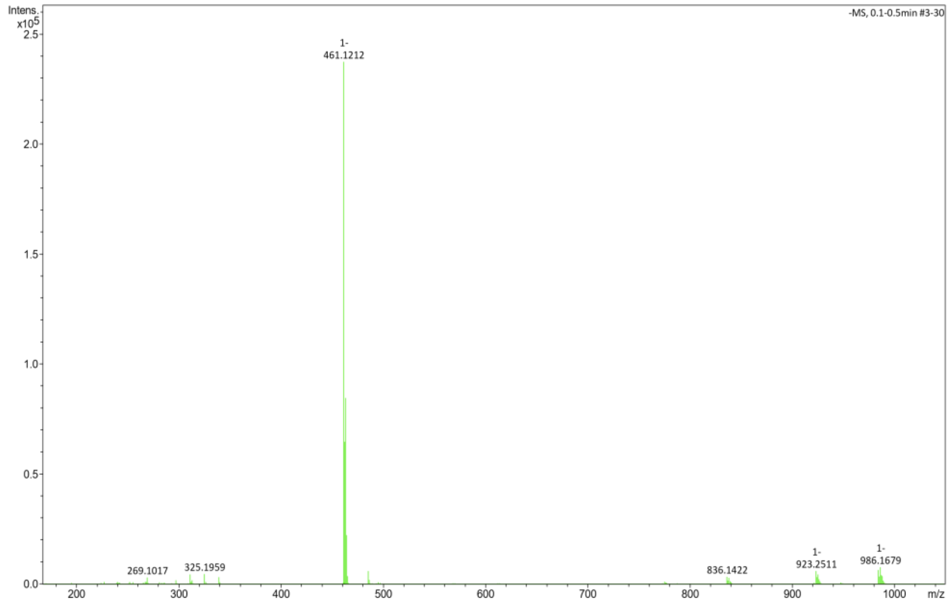

Figure S3. ESI-MS spectrum of compound **9d**

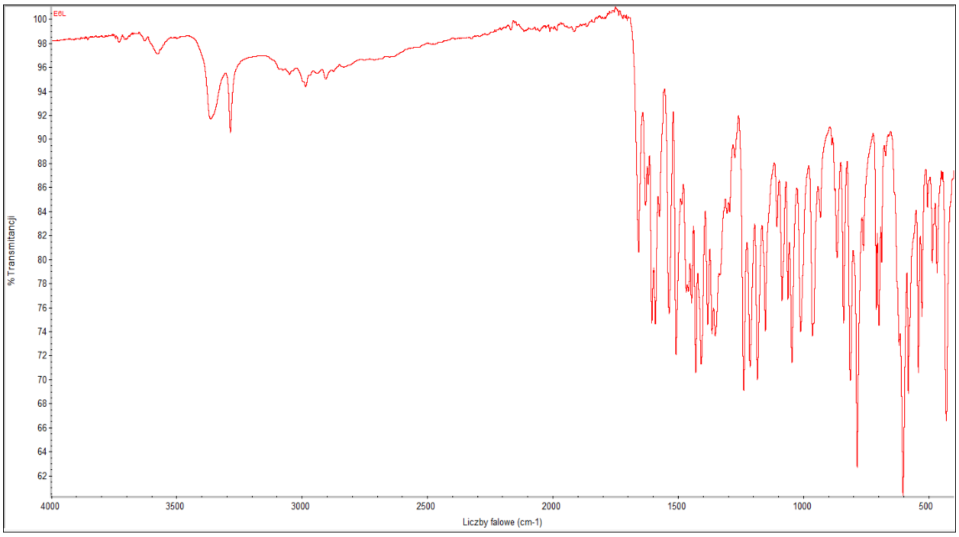

Figure S4. FT-IR spectrum of compound **9d**

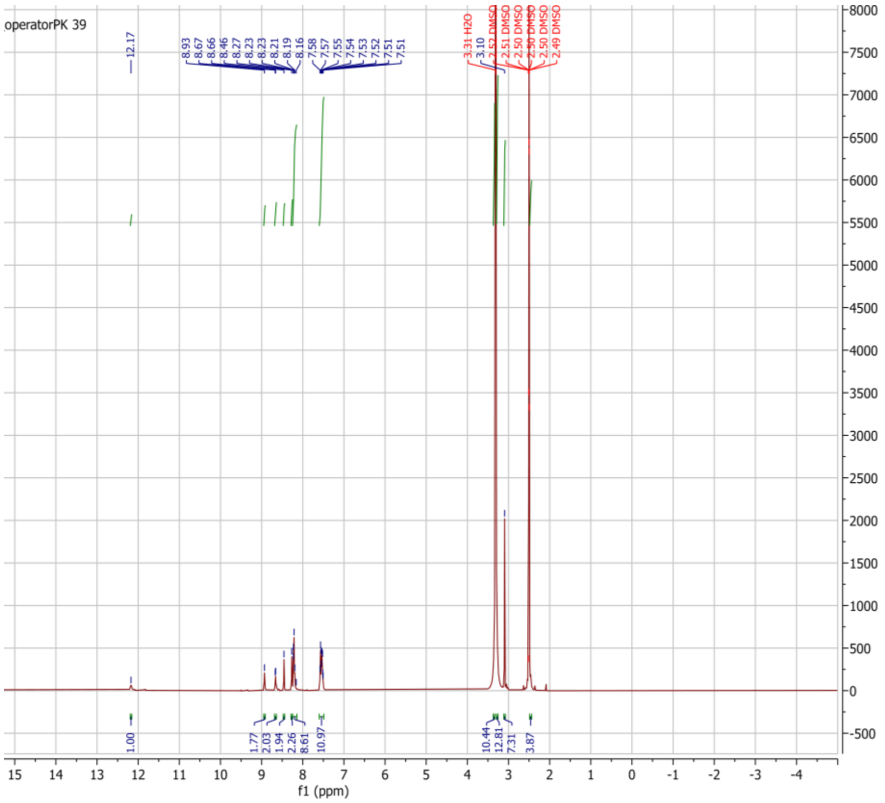

Figure S5. ^{1}H NMR spectrum of compound **10i**

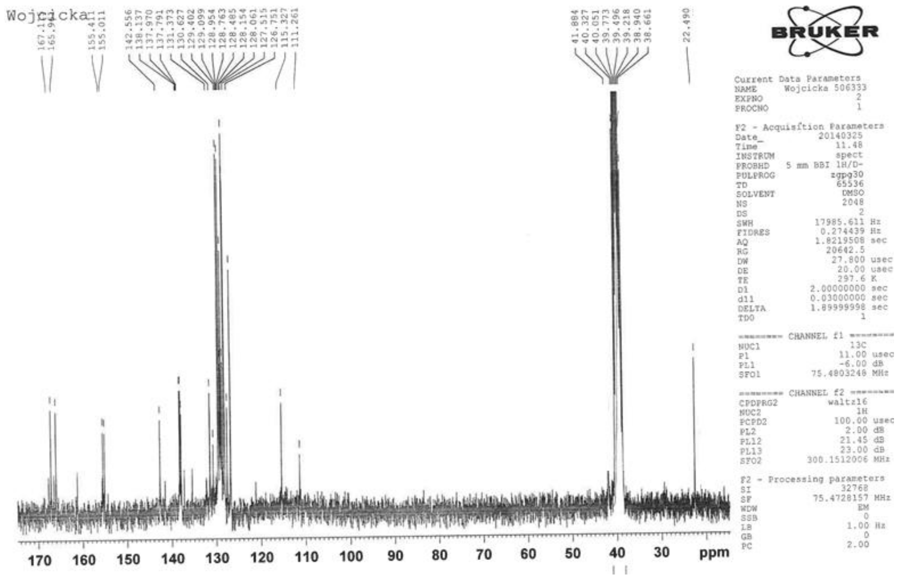

Figure S6. ^{13}C NMR spectrum of compound **10i**

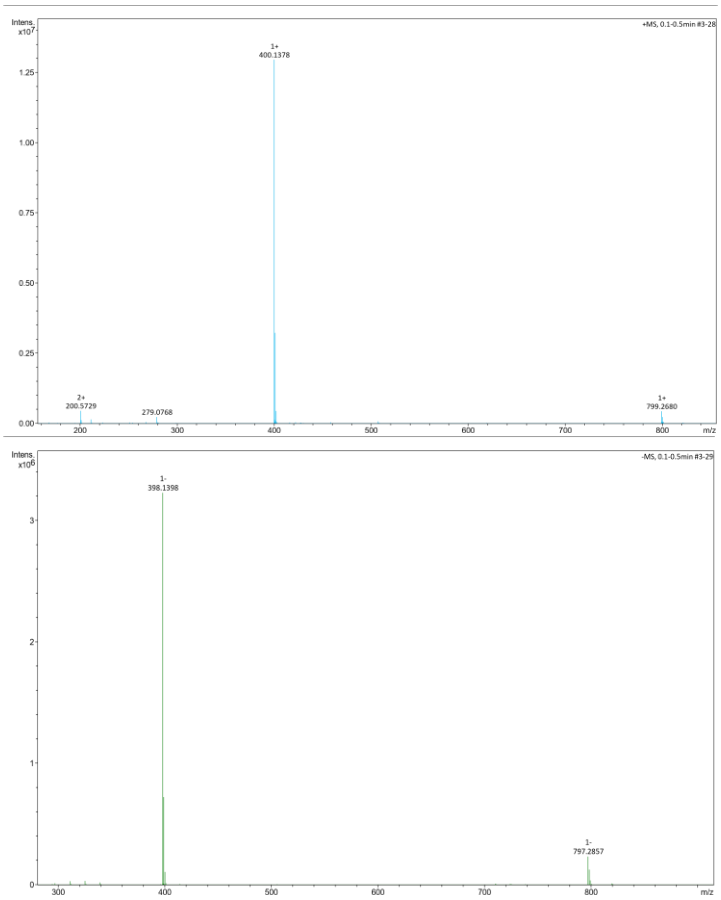

Figure S7. ESI-MS spectrum of compound **10i**

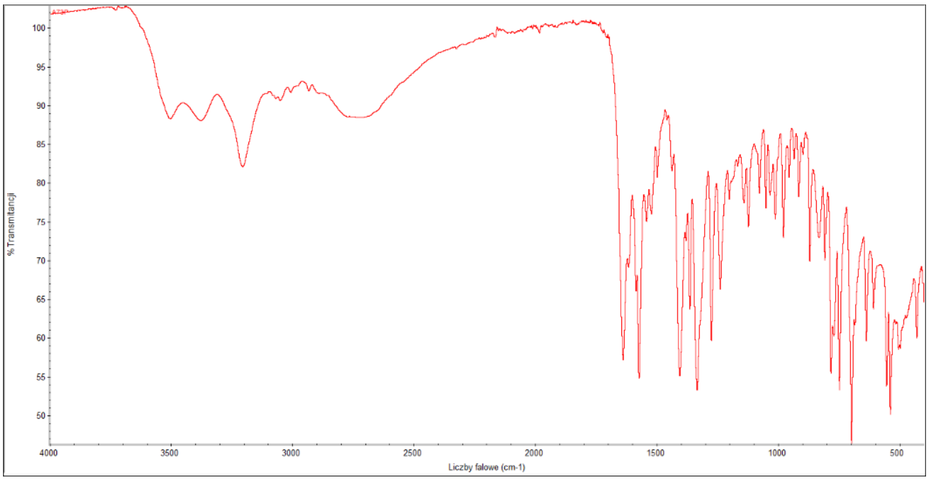

Figure S8. FT-IR spectrum of compound **10i**

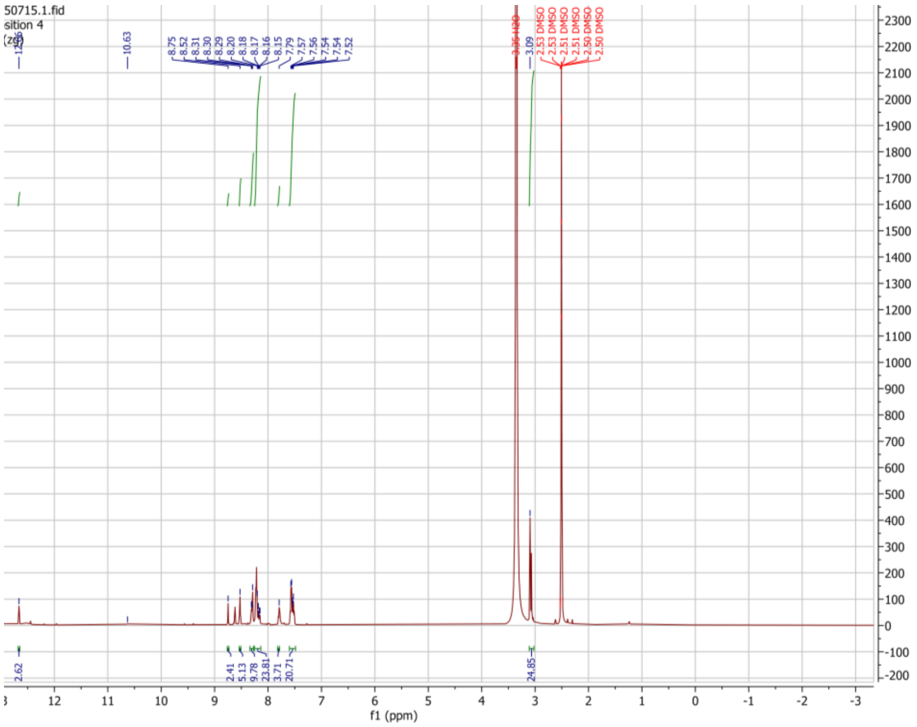

Figure S9. ^{1}H NMR spectrum of compound **10j**

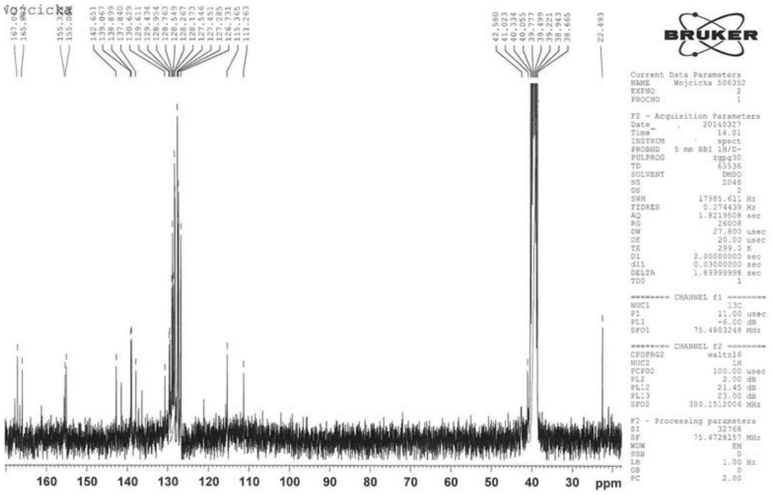

Figure S10. ^{13}C NMR spectrum of compound **10j**

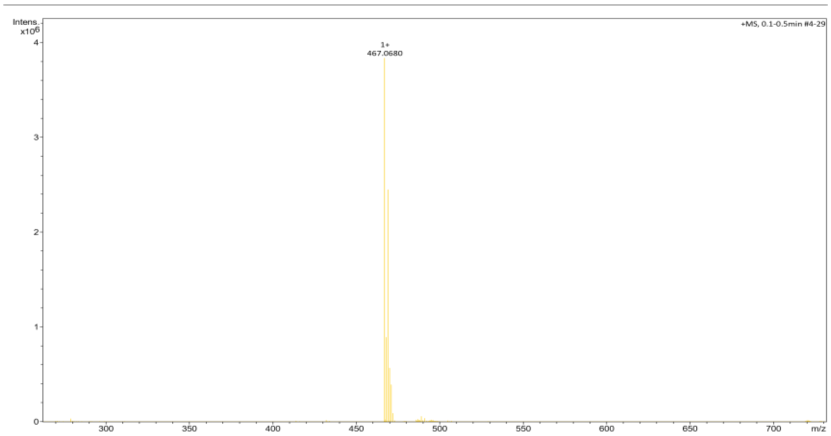

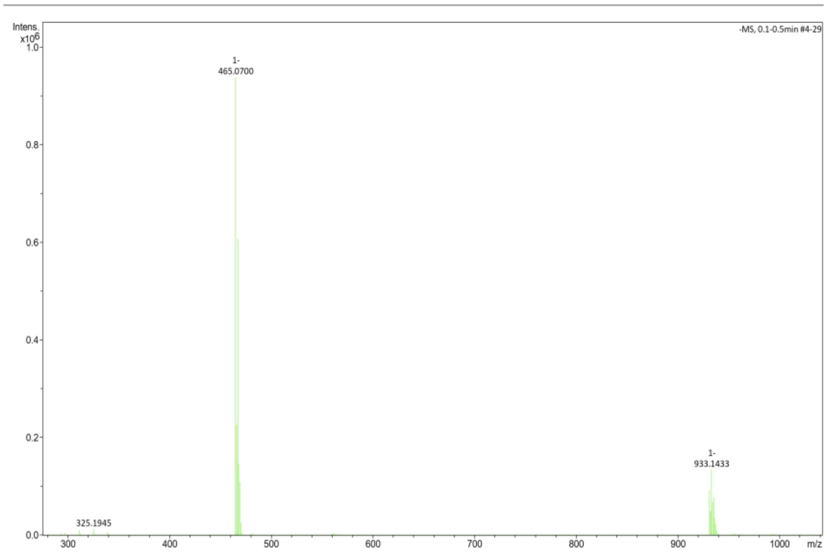

Figure S11. ESI-MS spectrum of compound **10j**

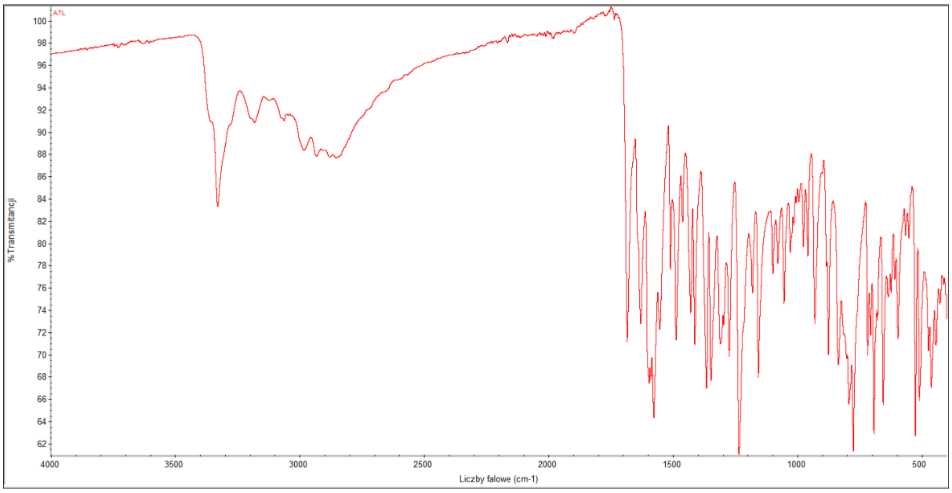

Figure S12. FT-IR spectrum of compound **10j**

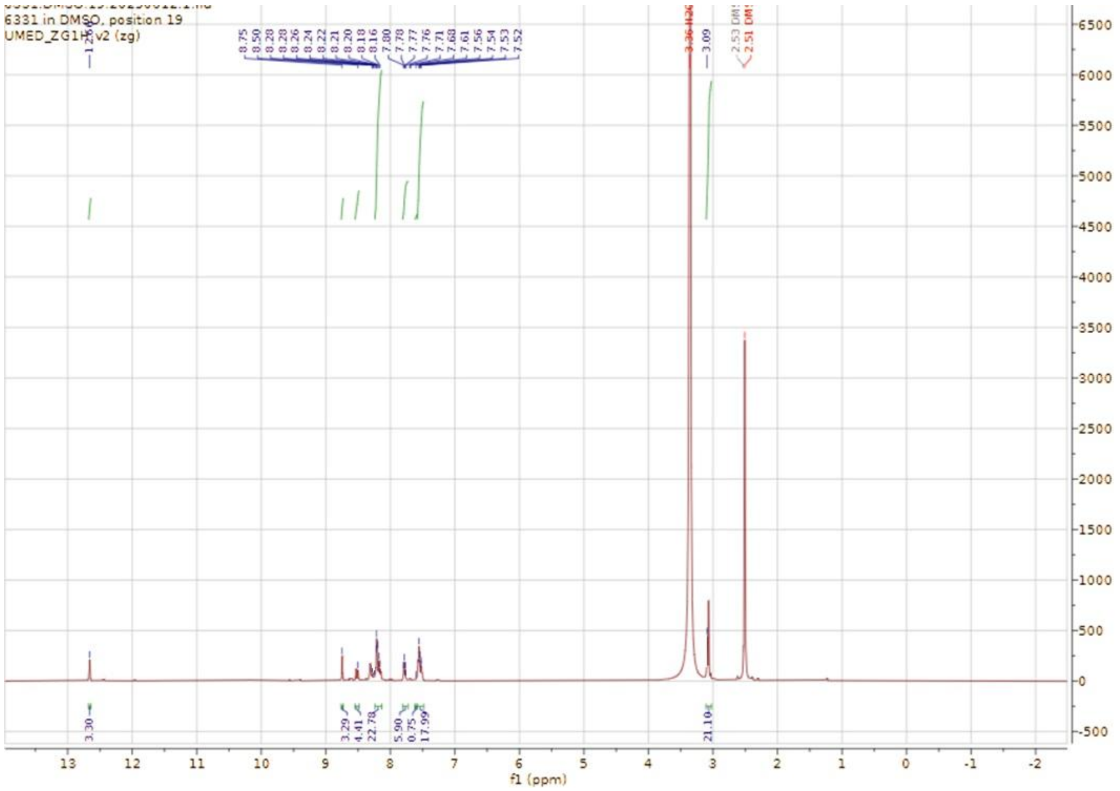

Figure S13. ^{1}H NMR spectrum of compound **11**

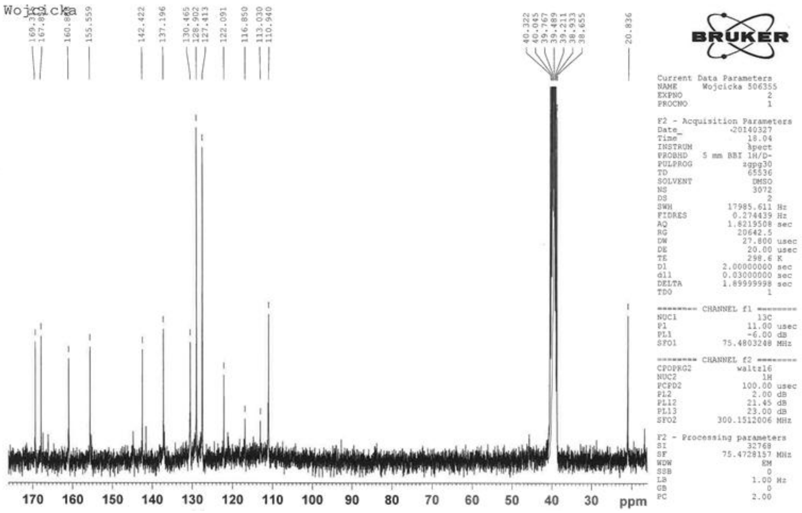

Figure S14. ^{13}C NMR spectrum of compound **11**

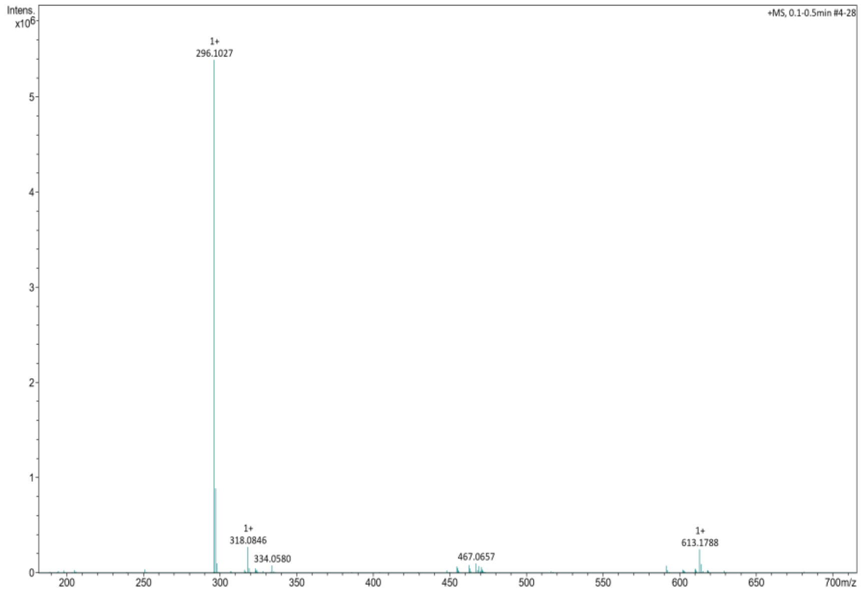

Figure S15. ESI-MS spectrum of compound **11**

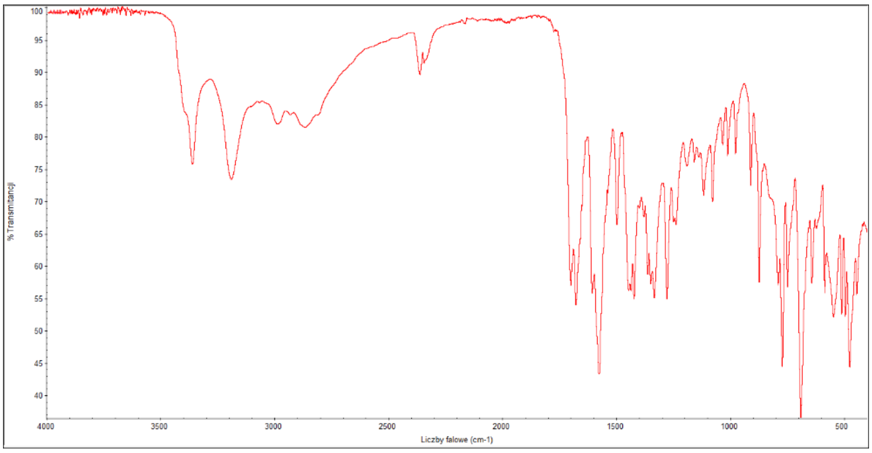

Figure S16. FT-IR spectrum of compound **11**

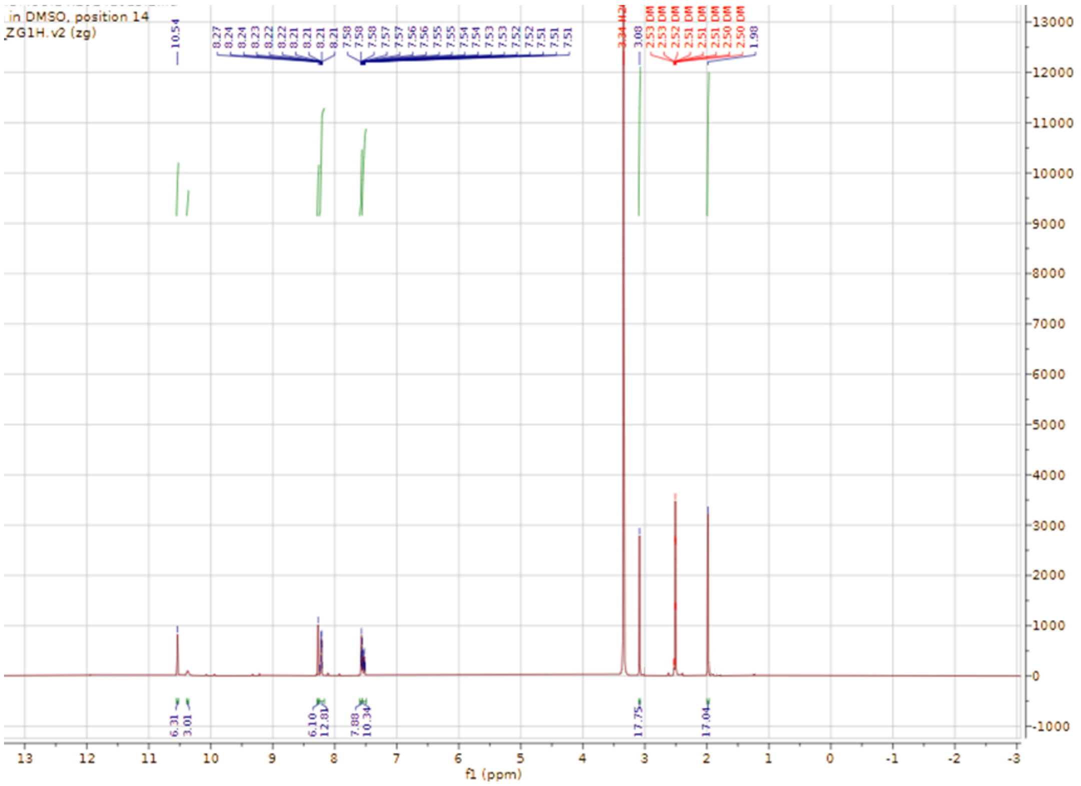

Figure S17. ^{1}H NMR spectrum of compound **12**

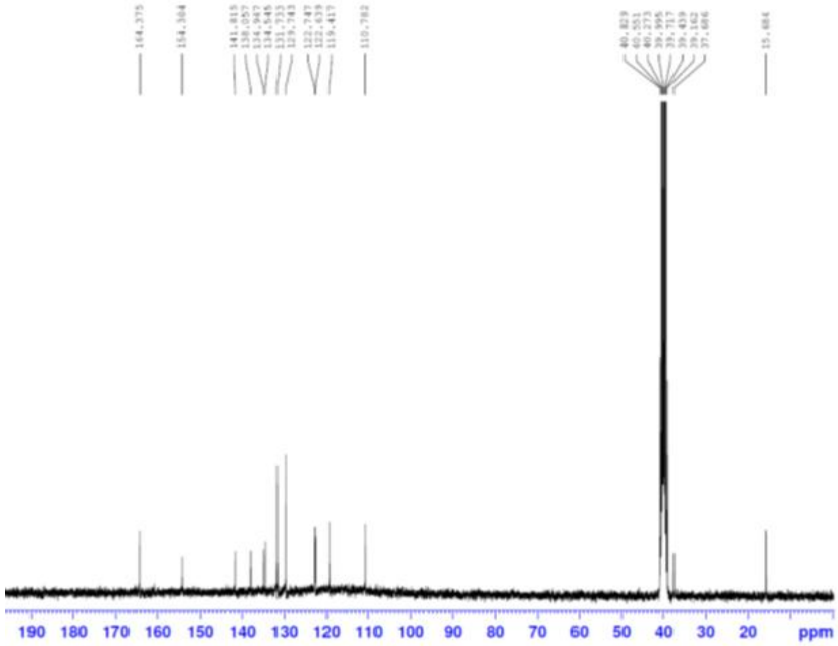

Figure S18. ^{13}C NMR spectrum of compound **12**

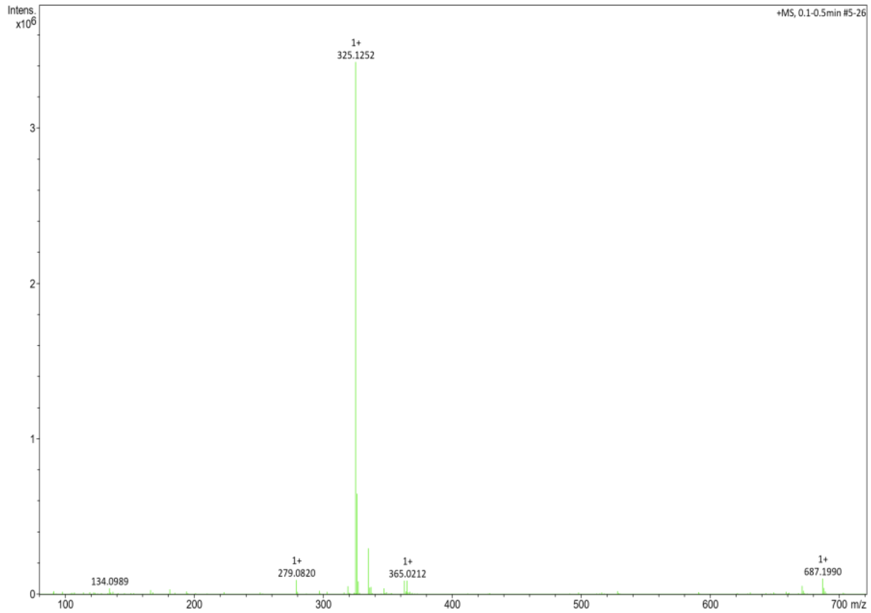

Figure S19. ESI-MS spectrum of compound **12**

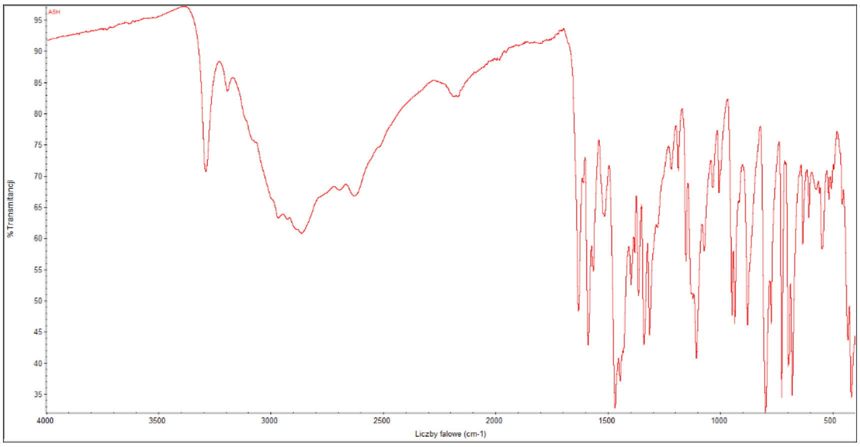

Figure S20. FT-IR spectrum of compound **12**

Table S1. Antimicrobial activity of Prontosan® (0.1% PHMB) expressed as minimal inhibitory concentration (MIC) and minimal bactericidal concentration (MBC) values determined by the 96-well microdilution method after 24 h incubation. The results demonstrate uniform bactericidal activity of PHMB against both Gram-positive and Gram-negative bacteria, with slightly higher concentrations required for *Candida albicans* and moderate susceptibility observed for *Lactobacillus crispatus*.

**microorganism**	**MIC [mg/L]**	**MBC [mg/L]**
S. aureus 29213	1	2
S. epidermidis 12228	2	2
Pseudomonas aeruginosa 115442	4	4
E. coli 10536	1	2
E. coli 8739	1	2
C.albicans 10231	4	8
L. crispatus 33197	4	8
